# A Fast Algorithm for Computing Binomial Coefficients Modulo Powers of Two

**DOI:** 10.1155/2013/751358

**Published:** 2013-11-06

**Authors:** Mugurel Ionut Andreica

**Affiliations:** Computer Science Department, Politehnica University of Bucharest, Splaiul Independentei 313, Sector 6, 060042 Bucharest, Romania

## Abstract

I present a new algorithm for computing binomial coefficients modulo 2^*N*^. The proposed method has an *O*(*N* ^3^ · Multiplication(*N*) + *N* ^4^) preprocessing time, after which a binomial coefficient *C*(*P*, *Q*) with 0 ≤ *Q* ≤ *P* ≤ 2^*N*^ − 1 can be computed modulo 2^*N*^ in *O*(*N* ^2^ · log(*N*) · Multiplication(*N*)) time. Multiplication(*N*) denotes the time complexity of multiplying two *N*-bit numbers, which can range from *O*(*N* ^2^) to *O*(*N* · log(*N*) · log(log(*N*))) or better. Thus, the overall time complexity for evaluating *M* binomial coefficients *C*(*P*, *Q*) modulo 2^*N*^ with 0 ≤ *Q* ≤ *P* ≤ 2^*N*^ − 1 is *O*((*N* ^3^ + *M* · *N* ^2^ · log(*N*)) · Multiplication(*N*) + *N* ^4^). After preprocessing, we can actually compute binomial coefficients modulo any 2^*R*^ with *R* ≤ *N*. For larger values of *P* and *Q*, variations of Lucas' theorem must be used first in order to reduce the computation to the evaluation of multiple (*O*(log*⁡*(*P*))) binomial coefficients *C*(*P*′, *Q*′) (or restricted types of factorials *P*′!) modulo 2^*N*^ with 0 ≤ *Q*′ ≤ *P*′ ≤ 2^*N*^ − 1.

## 1. Introduction

In this paper I present a novel efficient algorithm for computing binomial coefficients modulo 2^*N*^  (*N* ≥ 1). The definition of the binomial coefficient *C*(*P*, *Q*) is the usual one:

(1) $$\begin{array}{c} C\left(P,Q\right)=\frac{P!}{Q!\cdot \left(P-Q\right)!}. \end{array}$$

We will mainly consider the case where *P* ≤ 2^*N*^ − 1, and after fully handling this case, we will discuss how to compute *C*(*P*, *Q*) modulo 2^*N*^ for *P* ≥ 2^*N*^.

The presented algorithm consists of a preprocessing stage which takes *O*(*N* ^3^ · Multiplication(*N*) + *N* ^4^) time, where Multiplication(*N*) is the time complexity for multiplying two *N*-bit numbers. Multiplication(*N*) can range from *O*(*N* ^2^) (the naive multiplication algorithm) to *O*(*N* · log⁡⁡(*N*) · log⁡⁡(log⁡⁡(*N*))) or slightly better [1, 2].

After the preprocessing stage, a binomial coefficient *C*(*P*, *Q*) (with *P* ≤ 2^*N*^ − 1) can be computed modulo 2^*N*^ in *O*(*N* ^2^ · log⁡⁡(*N*) · Multiplication(*N*)) time. Of course, the presented algorithm can evaluate a binomial coefficient modulo any 2^*Q*^ with *Q* ≤ *N* (after computing it modulo 2^*N*^, we only need to keep the least significant *Q* bits out of the computed *N* bits).

The rest of this paper is structured as follows. In Sections 2–6 I present the steps of the preprocessing stage. In Sections 7–9 I present the algorithm which computes the binomial coefficients modulo 2^*N*^, considering that the preprocessing stage was completed. In Section 10 I discuss related work, and in Section 11 I conclude and discuss future work. I will summarize below the contents of each section describing the preprocessing steps and the algorithm itself.

The preprocessing stage consists of 5 steps. The first step consists of computing all the *small* binomial coefficients *C*(*P*, *Q*). The term *small* refers to the values of *P* and *Q*. A binomial coefficient *C*(*P*, *Q*) is defined to be *small* if 0 ≤ *Q* ≤ *P* ≤ *N*. This step is presented in Section 2. There are *O*(*N* ^2^)* small* binomial coefficients, and we can compute all of them with only *O*(*N* ^2^) additions of pairs of *N*-bit numbers.

The second preprocessing step (presented in Section 3) consists of computing a set of *large* binomial coefficients. All the binomial coefficients of the form *C*(2^*P*^ − *X*, *Q*), with 0 ≤ *P*, *X*, *Q* ≤ *N*, are defined to be *large*. There are *O*(*N* ^3^) such binomial coefficients and all of them can be computed in *O*(*N* ^2^ · Multiplication(*N*) + *N* ^4^) time overall. This step requires the largest amount of memory (among all the preprocessing steps) in order to store the *O*(*N* ^3^) large binomial coefficients.

In Section 4 I present the third step of the preprocessing stage, which consists of computing power sums of consecutive numbers (where the number of such numbers is a power of 2). There are *O*(*N* ^2^) power sums which can all be computed in *O*(*N* ^2^ · log⁡(*N*) · Multiplication(*N*)) time.

The 4th preprocesing step (presented in Section 5) consists of computing the sums of the products of the elements of all the subsets of a given size of a set consisting of the first 2^*P*^  (0 ≤ *P* ≤ *N*) positive integer numbers. In order to achieve this goal, inclusion-exclusion-based equations from the theory of elementary symmetric functions and polynomials are used. There are only *O*(*N* ^2^) values being computed, but it takes *O*(*N* ^3^ · Multiplication(*N*)) time to compute them. This step is the performance bottleneck step in the preprocessing stage.

Finally, in Section 6 I present the last step of the preprocessing stage, computing the sums of the products of the elements of all the odd-element subsets of a given size of a set consisting of the first 2^*P*^  (0 ≤ *P* ≤ *N*) positive integer numbers. Like in the previous case there are *O*(*N* ^2^) values which need to be computed during this step.

In Section 7 I will show how to efficiently find the largest odd divisor (modulo 2^*N*^) of *P*! (for *P* ≤ 2^*N*^ − 1). In Section 8 I will present the actual algorithm for computing binomial coefficients *C*(*P*, *Q*) modulo 2^*N*^ (for *P* ≤ 2^*N*^ − 1), and in Section 9 I will discuss extensions to the case *P* ≥ 2^*N*^.

Note that by precomputing all the mentioned values, we are capable of achieving a running time of *O*(*N* ^2^ · log⁡(*N*) · Multiplication(*N*)) for computing a single binomial coefficient *C*(*P*, *Q*) (with 0 ≤ *Q* ≤ *P* ≤ 2^*N*^ − 1). When computing *M* binomial coefficients we achieve a running time of *O*(*M* · *N* ^2^ · log⁡(*N*) · Multiplication(*N*)). Since computing a binomial coefficient requires the values from the preprocessing stage, the time complexity would increase significantly if we had to compute all those values for each binomial coefficient (instead of computing them only once and then reusing them for each binomial coefficient).

## 2. Computing Small Binomial Coefficients: *C*(*P*, *Q*) for 0 ≤ *Q* ≤ *P* ≤ *N*

The first step of the preprocessing algorithm consists of computing the binomial coefficients SC(*P*, *Q*) = *C*(*P*, *Q*)mod⁡ 2^*N*^ for *small* values of *P* and *Q*  (0 ≤ *Q* ≤ *P* ≤ *N*). This can be achieved easily with *O*(*N* ^2^)  *N*-bit additions (*O*(*N* ^3^) time overall, as an addition takes *O*(*N*) time). We have

(2) $$\begin{array}{c} \text{SC}\left(P,0\right)=1,\\ \text{SC}\left(P,Q>P\right)=0. \end{array}$$

For 1 ≤ *Q* ≤ *P*, we use the well-known formula:

(3) SC(P,Q)=SC(P−1,Q−1)+SC(P−1,Q) (mod⁡ 2N).

## 3. Computing Large Binomial Coefficients: *C*(2^*P*^ − *X*, *Q*) for 0 ≤ *P*, *X*, *Q* ≤ *N*

The second step of the preprocessing algorithm consists of computing *large* binomial coefficients *C*(2^*P*^ − *X*, *Q*) modulo 2^*N*^ for 0 ≤ *P*, *X*, *Q* ≤ *N*; we denote these values by LC(*P*, *X*, *Q*). Obviously, if 2^*P*^ < *X* or *Q* > 2^*P*^ − *X*, then LC(*P*, *X*, *Q*) = 0. Otherwise, let

(4) $$\begin{array}{c} X\max\left(P\right)=\min\left\{2^{P},N\right\}. \end{array}$$

For 0 ≤ *X* ≤ *X*max⁡⁡(*P*), from the definition of the binomial coefficients, we have that

(5) $$\begin{array}{c} \text{LC}\left(P,X,0\right)=1,\\ \text{LC}\left(P,X,Q>0\right)=\text{LC}\left(P,X,Q-1\right)\cdot \frac{\left(2^{P}-X-Q+1\right)}{Q}. \end{array}$$

Since all the computations are performed modulo 2^*N*^, we need to perform multiplications by *Q* ^−1^. But *Q* only has a multiplicative inverse (modulo 2^*N*^) if it is odd. Thus, we will have to compute two different values:

- LC_2_(*P*, *X*, *Q*) = the largest odd divisor of *C*(2^*P*^ − *X*, *Q*);
- Exp_2_(*P*, *X*, *Q*) = the exponent of 2 in the prime factor decomposition of *C*(2^*P*^ − *X*, *Q*).

We start with LC_2_(*P*, *X*, 0) = 1 and Exp_2_(*P*, *X*, 0) = 0. For *Q* > 0, we will compute the following:

- *A* = the largest odd divisor of 2^*P*^ − *X* − *Q* + 1,
- *B* = the exponent of 2 in the prime factor decomposition of 2^*P*^ − *X* − *Q* + 1,
- *C* = the largest odd divisor of *Q*,
- *D* = the exponent of 2 in the prime factor decomposition of *Q*.

Finding *A* and *B* can be performed in *O*(*N*) time by examining the bits of 2^*P*^ − *X* − *Q* + 1. With these values computed we will have

- LC_2_(*P*, *X*, *Q* > 0) = LC_2_(*P*, *X*, *Q* − 1) · *A* · *C* ^−1^;
- Exp_2_(*P*, *X*, *Q* > 0) = Exp_2_(*P*, *X*, *Q*) + *B* − *D*.

We will assume that the multiplicative inverses of all the odd numbers *C* from 1 to *N* were precomputed. The inverse of an odd number *C* (modulo 2^*N*^, for *N* ≥ 3) is equal to

(6) $$\begin{array}{c} C^{-1}=C^{2^{N-2}-1}. \end{array}$$

For *N* ≤ 2, the inverse of an odd number is the odd number itself. Equation (6) provides a way of computing the inverse of an odd number *C* < 2^*N*^ using *O*(*N*)  *N*-bit multiplications. Note that more efficient alternatives exist; for example, in [3], an algorithm with *O*(*N*)  *N*-bit additions, left bit-shifts, and right bit-shifts for computing the inverse is presented (which would take only *O*(*N* ^2^) time overall instead of *O*(*N* · Multiplication(*N*))). However, the algorithm obtained from (6) will never be the bottleneck in any step of the presented algorithm, so there will be no problem using it.

After computing the values LC_2_(*P*, *X*, *Q*), and Exp_2_(*P*, *X*, *Q*) we have

(7) LC(P,X,Q)  =LC2(P,X,Q)·2Exp2(P,X,Q) (mod⁡ 2N).

We will assume that we previously precomputed all the numbers 2^*j*^ for 0 ≤ *j* ≤ *N*. This way we have a method for computing all the values LC(*P*, *X*, *Q*).

Let us perform a time complexity analysis. For each of the *O*(*N* ^3^) tuples (*P*, *X*, *Q*), we spend *O*(*N* + Multiplication(*N*)) time. Precomputing the inverses of *small* numbers *Q*  (0 ≤ *Q* ≤ *N*) takes *O*(*N* ^2^ · Multiplication(*N*)) time (it takes *O*(*N* · Multiplication(*N*)) time to apply (6)). Precomputing the powers of two takes only *O*(*N* ^2^) time (we simply shift 2^*j*^ one bit to the left to obtain 2^*j*+1^).

A more efficient method is to apply the previously defined algorithm only for *X* = *X*max⁡(*P*). Then, for 0 ≤ *X* < *X*max⁡(*P*) and 1 ≤ *Q* ≤ min⁡{*N*, 2^*P*^ − *X*}, we have

(8) LC(P,X,Q)=LC(P,X+1,Q−1) +LC(P,X+1,Q) (mod⁡ 2N).

With this method, we spend *O*(*N* + Multiplication(*N*)) time only for *O*(*N* ^2^) tuples (the tuples (*P*, *X*, 0)). For the remaining *O*(*N* ^3^) tuples, we perform a simple addition (which takes *O*(*N*) time). Thus, the overall time complexity is *O*(*N* ^2^ · Multiplication(*N*) + *N* ^4^).

## 4. Computing Power Sums of Consecutive Numbers

In this section we will efficiently compute power sums of consecutive numbers starting from 1 and ending at a power of two. We define

(9) PSUM(P,Q)=1Q+2Q+⋯+(2P)Q=∑k=12PkQ (mod⁡ 2N),

where 0 ≤ *P* ≤ *N* and 0 ≤ *Q* ≤ *N*.

We will start with the easy cases: PSUM(0, *Q*) = 1 (independent of the value of *Q*) and PSUM(*P*, 0) = 2^*P*^  (mod⁡ 2^*N*^).

For *P* ≥ 1 and *Q* ≥ 1, we can write

(10) PSUM(P,Q)=PSUM(P−1,Q) +(2P−1+1)Q+(2P−1+2)Q +⋯+(2P−1+2P−1)Q (mod⁡ 2N).

Let us consider the terms (2^*P*−1^ + *i*)^*Q*^ (1 ≤ *i* ≤ 2^*P*−1^). Using Newton's binomial theorem, we can write this term as

(11) $$\begin{array}{c} \overset{Q}{\underset{k=0}{\sum }}C\left(Q,k\right)\cdot {\left(2^{P-1}\right)}^{k}\cdot i^{Q-k}. \end{array}$$

Thus, PSUM(*P*, *Q*) can be written as

(12) PSUM(P−1,Q)+∑i=12P−1∑k=0QC(Q,k)·(2P−1)k·iQ−k  =∑k=0QC(Q,k)·(2P−1)k·∑i=12P−1iQ−k  =∑k=0QC(Q,k)·(2P−1)k·PSUM(P−1,Q−k).

Note that all the terms with *k* > *N*/(*P* − 1) are zero modulo 2^*N*^ (because 2^(*P*−1)·*k*^ is a multiple of 2^*N*^). Thus, we only need to evaluate *O*(min⁡{*Q*, *N*/(*P* − 1)}) such terms:

(13) PSUM(P,Q)=PSUM(P−1,Q) +∑k=0min⁡⁡{Q,N/(P−1)}SC(Q,k)·2(P−1)·k·PSUM(P−1,Q−k).

Assuming that we previously computed all the values SC(*i*, *j*)  (0 ≤ *i*, *j* ≤ *N*), then this part takes *O*(*N* ^2^ · log⁡(*N*) · Multiplication(*N*)) time (because, for each value of *Q*, the algorithm performs at most *O*(*Q* + *N*/1 + *N*/2 + ⋯+*N*/(*N* − 1)) = *O*(*N* · log⁡(*N*)) multiplications for all the values of *P*).

## 5. Computing Sums of Subset Products

In this section we will compute the values SSP(*P*, *Q*) = the sum of all the products of the elements of the *Q*-element subsets of the numbers 1,2,…, 2^*P*^ (modulo 2^*N*^) for 0 ≤ *P* ≤ *N* and 0 ≤ *Q* ≤ min⁡{2^*P*^, *N*}. To be more precise,

(14) SSP(P,Q) =∑1≤v(1)<v(2)<⋯<v(Q)≤2Pv(1)· ⋯ ·v(Q) (mod⁡ 2N).

We have SSP(*P*, 0) = 1 and SSP(*P*, 1) = PSUM(*P*, 1) (i.e., the sum of all the numbers 1, 2,…, 2^*P*^).

For 2 ≤ *Q* ≤ *N*, we will use formulas based on the inclusion-exclusion principle derived from the theory of power sum and elementary symmetric polynomials [4]:

(15) $$\begin{array}{c} \text{SSP}\left(P,Q\right)=Q^{-1}\cdot \overset{Q}{\underset{k=1}{\sum }}{\left(-1\right)}^{k-1}\cdot \text{SSP}\left(P,Q-k\right)\cdot \text{PSUM}\left(P,k\right). \end{array}$$

The problem that we face now is that *Q* ^−1^(mod⁡ 2^*N*^) exists only when *Q* is odd. This implies that we will not be able to compute all the values SSP(*P*, *Q*) (for a fixed value of *P*) with the same precision. Let us define Precision(*P*, *Q*) = the maximum value *U* ≤ *N* such that the last *U* bits of SSP(*P*, *Q*) are correct (i.e., we were able to compute SSP(*P*, *Q*) modulo 2^*U*^ but not modulo 2^*V*^ for *V* > *U*, unless we perform computations modulo 2^*N*′^ for *N*′ > *N*). Obviously, Precision(*P*, 0) = Precision(*P*, 1) = *N*.

Let us consider the case *Q* ≥ 2. We will have Precision(*P*, *Q*) ≤ Precision(*P*, *Q* − 1): the precision either stays the same or decreases as *Q* increases. At first we evaluate the following sum:

(16) Sum(P,Q)  =∑k=1Q(−1)k−1·SSP(P,Q−k)·PSUM(P,k).

This sum is correctly computed modulo 2^Precision(*P*,*Q*−1)^. Then we compute the following:

- *A* = the largest odd divisor of *Q*;
- *B* = the exponent of 2 in the prime factor decomposition of *Q*.

We set Precision(*P*, *Q*) = Precision(*P*, *Q* − 1) − *B*. We then multiply Sum(*P*, *Q*) by *A* ^−1^, obtaining Sum′(*P*, *Q*). Finally, we can compute SSP(*P*, *Q*) as Sum′(*P*, *Q*) divided by 2^*B*^. It should be mentioned that *A* ^−1^ is computed as the multiplicative inverse of *A* modulo 2^Precision(*P*,*Q*−1)^ instead of modulo 2^*N*^.

We should now prove that Precision(*P*, *Q*) > *N* − *Q* for *Q* ≥ 1. This is obviously true for *Q* = 1. In general, Precision(*P*, *Q*) is equal to *N* minus the exponent of 2 in *Q*!. Since the exponent of 2 in *Q*! is equal to

(17) $$\begin{array}{c} \overset{\text{lo}{\text{g}}_{2}Q}{\underset{k=1}{\sum }}\frac{Q}{2^{k}}, \end{array}$$

it is obvious that this value cannot exceed *Q* − 1. Thus, Precision(*P*, *Q*) ≥ *N* − (*Q* − 1) > *N* − *Q*. This proof is very important. Although we cannot compute all the SSP(*P*, *Q*) values with the same precision, we will see that this precision will be sufficient in order to obtain exact and correct results when computing binomial coefficients modulo 2^*N*^. This is because in all the equations where SSP(*P*, *Q*) is involved, it will be multiplied by 2^*Q*^.

Let us perform the time complexity analysis now. For each pair (*P*, *Q*), we need to perform *N* multiplication of *N*-bit numbers. It would seem that we also need to compute *Q* ^−1^ for each pair (*P*, *Q*) (which would require another *N* multiplications of *N*-bit numbers for raising *Q* to the appropriate power). Although this would not affect the theoretical time complexity, we should notice that Precision(*P*, *Q*) depends only on the numbers 1,…, *Q* and does not depend on *P* at all (we only need to have *Q* ≤ 2^*P*^). Thus, we only need to compute *Q* ^−1^ once for the first value of *P* for which we will compute SSP(*P*, *Q*) (and then we will cache *Q* ^−1^ and use it later when it is needed). Overall, the time complexity is dominated by the *N* ^3^ multiplications which need to be performed, obtaining an *O*(*N* ^3^ · Multiplication(*N*)) time complexity.

This step of the preprocessing stage is the bottleneck, having the largest time complexity (it is the step which dominates the computations of the preprocessing stage).

## 6. Computing Sums of Odd-Element Subset Products

In this section we will compute the values SOSP(*P*, *Q*) = the sum of all the products of the elements of the odd *R*-element subsets of the numbers 1,2,…, 2^*P*^, where *R* = 2^*P*−1^ − *Q* (for 1 ≤ *P* ≤ *N* and 0 ≤ *Q* ≤ min⁡{2^*P*−1^, *N*}). To be more precise,

(18) SOSP(P,Q) =∑1≤u(1)<u(2)<⋯<u(R)<2Pu(i) is odd,1≤i≤RR=2P−1−Qu(1)· ⋯ ·u(R) (mod⁡ 2N).

This time we are interested in subsets with large sizes, that is, sizes ranging from 2^*P*−1^ − *N* to 2^*P*−1^ (thus having 0 ≤ *Q* ≤ *N*).

For *Q* = 0, we are interested in computing the product

(19) $$\begin{array}{c} \overset{2^{P-1}}{\underset{k=1}{\prod }}\left(2\cdot k-1\right)\;\left(\mathrm{mod}\;2^{N}\right). \end{array}$$

By using Newton's binomial theorem, we can rewrite (19) as

(20) $$\begin{array}{c} \overset{2^{P-1}}{\underset{j=0}{\sum }}2^{j}\cdot \text{SSP}\left(P-1,j\right)\cdot {\left(-1\right)}^{2^{P-1}-j}\;\left(\mathrm{mod}\;2^{N}\right). \end{array}$$

We notice that, for *j* ≥ *N*, each term of the sum is zero modulo 2^*N*^. Moreover, notice that SSP(*P* − 1, *j*) is multiplied by 2^*j*^. Since the precision of SSP(*P* − 1, *j*) is larger than *N* − *j*, the result of this multiplication is exact modulo 2^*N*^. Thus, by iterating through all the values of *j* from 0 to min⁡{2^*P*−1^, *N* − 1}, we have an algorithm which requires *O*(*N*) multiplications of *N*-bit numbers for computing SOSP(*P*, 0).

For *Q* > 0, we will need to use a different approach. Let us choose a subset of odd numbers from 1 to 2^*P*−1^ of size 2^*P*−1^ − *Q*: *u*(1),…, *u*(2^*P*−1^ − *Q*). The product of all the elements in the subset is equal to *u*(1) ·  ⋯ ·*u*(2^*P*−1^ − *Q*) = (2 · *v*(1) − 1)·⋯·(2 · *v*(2^*P*−1^ − *Q*) − 1) (we wrote *u*(*i*) = 2 · *v*(*i*) − 1 for 1 ≤ *i* ≤ 2^*P*−1^ − *Q*). By using Newton's binomial theorem, we can rewrite this product as

(21) $$\begin{array}{c} \overset{2^{P-1}-Q}{\underset{j=0}{\sum }}{\left(-1\right)}^{2^{P-1}-Q-j}\cdot 2^{j}\cdot \text{SSP}{\left(P-1,j\right)}_{\;|\;v(1),\ldots ,v(2^{P-1}-Q)}, \end{array}$$

where we denoted by SSP(*P*−1,*j*)_∣*v*(1),…,*v*(2^*P*−1^−*Q*)_ the sum of products of the elements of the subsets of size *j* of the set {*v*(1),…, *v*(2^*P*−1^ − *Q*)}. In order to compute SOSP(*P*, *Q*), we will need to sum (21) over all the odd-element subsets *u*(1),…, *u*(2^*P*−1^ − *Q*) (or, equivalently, over all the subsets *v*(1),…, *v*(2^*P*−1^ − *Q*) of the set {1, 2,…, 2^*P*−1^}). Again, we should notice that all the terms of the sum from (21) are zero modulo 2^*N*^ for *j* ≥ *N*.

Let us consider a subset *w*(1),…, *w*(*j*) of the set {1,…, 2^*P*−1^} (with 0 ≤ *j* ≤ *N*). This subset is part of *C*(2^*P*−1^ − *j*, 2^*P*−1^ − *Q* − *j*) subsets *v*(1),…, *v*(2^*P*−1^ − *Q*) (where each element is chosen from the set {1,…, 2^*P*−1^}). With this observation we can rewrite (21) as

(22) ∑j=0min⁡{N−1,2P−1−Q}(−1)2P−1−Q−j·2j  ·C(2P−1−j,2P−1−Q−j)·SSP(P−1,j).

Since *C*(2^*P*−1^ − *j*, 2^*P*−1^ − *Q* − *j*) = *C*(2^*P*−1^ − *j*, *Q*) and we already computed these values as LC(*P* − 1, *j*, *Q*), we can finally rewrite (22) as

(23) ∑j=0min⁡{N−1,2P−1−Q}(−1)2P−1−Q−j·2j  ·LC(P−1,j,Q)·SSP(P−1,j).

We can now use (23) directly in order to obtain an algorithm performing *O*(*N*) multiplications of *N*-bit numbers for computing SOSP(*P*, *Q*). Of course, as before, all computations are performed modulo 2^*N*^. The time complexity in this case is simple to analyze: there are *O*(*N* ^3^) multiplications performed, so the complexity is *O*(*N* ^3^ · Multiplication(*N*)).

This part can be improved because, as we will see in Section 7, for a given value of *P*, we will only need the values SOSP(*P*, *Q*) such that 0 ≤ *Q* ≤ *N*/(*P* + 1). Thus, overall, we actually need to compute the values SOSP(*P*, *Q*) for only *O*(*N* · log⁡(*N*)) pairs (*P*, *Q*) instead of *O*(*N* ^2^) such pairs. In this case the time complexity drops down to *O*(*N* ^2^ · log⁡(*N*) · Multiplication(*N*)).

## 7. Computing the Largest Odd Divisor of *P*! Modulo 2^*N*^

In this section we will compute the largest odd divisor of *P*! (modulo 2^*N*^). We will denote this number by *F*_2_(*P*). Let us consider the binary decomposition of *P*; that is,

(24) $$\begin{array}{c} P=2^{Q(1)}+\cdots +2^{Q(K)}, \end{array}$$

where *Q*(1)>⋯>*Q*(*K*) (we will assume that *P* ≥ 1, and thus, *K* ≥ 1).

We will first evaluate FODD(*P*) = the product of all the odd numbers less than or equal to *P*!  (modulo  2^*N*^). For each bit *Q*(*i*) of *P*, we will compute a value FFODD(*P*, *i*). FFODD(*P*, 1) will be equal to the product (modulo  2^*N*^) of all the odd numbers from the interval [1,2^*Q*(1)^]. FFODD(*P*, *i* > 1) will be equal to the product (modulo  2^*N*^) of all the odd numbers from the interval [2^*Q*(1)^ + ⋯+2^*Q*(*i*−1)^ + 1, 2^*Q*(1)^ + ⋯ + 2^*Q*(*i*−1)^ + 2^*Q*(*i*)^]. Thus, we will have

(25) $$\begin{array}{c} \text{FODD}\left(P\right)=\text{FFODD}\left(P,1\right)+\cdots +\text{FFODD}\left(P,K\right). \end{array}$$

FFODD(*P*, 1) is easy to compute. If *Q*(1) = 0, then FFODD(*P*, 1) = 1; otherwise it is equal to SOSP(*Q*(1), 0).

Let us consider now the case *i* > 1. Let *X*(*i*) be equal to 2^*Q*(1)^ + ⋯+2^*Q*(*i*−1)^. If *Q*(*i*) = 0, then FFODD(*P*, *i*) = (*X*(*i*) + 1) mod⁡ 2^*N*^. Otherwise we can write FFODD(*P*, *i*) as

(26) $$\begin{array}{c} \overset{2^{Q(i)-1}}{\underset{j=1}{\prod }}\left(X\left(i\right)-1+2\cdot j\right)\;\left(\mathrm{mod}\;2^{N}\right). \end{array}$$

By using Newton's binomial theorem, we can rewrite (26) as

(27) $$\begin{array}{c} \overset{2^{Q(i)-1}}{\underset{j=0}{\sum }}2^{j}\cdot \text{SSP}\left(Q\left(i\right)-1,j\right)\cdot {\left(X\left(i\right)-1\right)}^{2^{Q(i)-1}-j}. \end{array}$$

We must notice that we only need to consider values of *j* up to min⁡{2^*Q*(*i*)−1^, *N* − 1}, because for *j* ≥ *N*, the corresponding term of the sum is zero modulo 2^*N*^. We should also notice that SSP(*Q*(*i*) − 1, *j*) is multiplied by 2^*j*^. Since its precision is larger than *N* − *j*, the multiplication of SSP(*Q*(*i*) − 1, *j*) and 2^*j*^ is exact modulo 2^*N*^. Then we iterate with *j* in descending order. For the initial value of *j*, we compute *XP*(*i*) = (*X*(*i*)−1)^*j*^; for the other values, we only multiply *XP*(*i*) by (*X*(*i*)−1)^−1^ in order to obtain *XP*(*i*) as (*X*(*i*)−1)^*j*^. We now have an algorithm performing *O*(*N*) multiplications of *N*-bit numbers for computing FFODD(*P*, *i*). Overall, we have an algorithm performing *O*(*N* ^2^) multiplications of *N*-bit numbers for computing FODD(*P*) (*O*(*N*) multiplications for each of the *O*(*N*) bits of *P*). However, we can do better by rewriting (26) in a different way:

(28) $$\begin{array}{c} \overset{2^{Q(i)-1}}{\underset{j=0}{\sum }}X{\left(i\right)}^{j}\cdot \text{SOSP}\left(Q\left(i\right),j\right). \end{array}$$

Note that *X*(*i*) is even. In fact, it is a multiple of 2^*Q*(*i*−1)^, meaning that it is at least a multiple of 2^*Q*(*i*)+1^. For *j* > (*N*/(*Q*(*i*) + 1)), each term of the sum will be zero modulo 2^*N*^. Thus, we only need to consider at most *N*/(*Q*(*i*) + 1) + 1 terms (from *j* = 0 to *j* = *N*/(*Q*(*i*) + 1)). We will start with *XP*(*i*) = 1 when *j* = 0, and then for each subsequent value of *j*, we will multiply *XP*(*i*) by *X*(*i*) in order to have *XP*(*i*) = *X*(*i*)^*j*^ at each iteration.

Overall we only need to consider *O*(*N* · log⁡(*N*)) terms for computing FODD(*P*)(*O*(*N* + *N*/(*Q*(2) + 1) + *N*/(*Q*(3) + 1)+⋯+*N*/(*Q*(*K*) + 1))); for each such term, an *N*-bit multiplication needs to be performed.

Now that we have a method of efficiently computing FODD(*P*), we can use it in order to write *F*_2_(*P* > 1) as

(29) $$\begin{array}{c} F_{2}\left(P>1\right)=\text{FODD}\left(P\right)+F_{2}\left(\frac{P}{2}\right). \end{array}$$

*F*_2_(0 ≤ *P* ≤ 1) is equal to 1. Since *P* is of the order of magnitude of 2^*N*^ and FODD(*P*) can be evaluated with *O*(*N* · log⁡(*N*))  *N*-bit multiplications, computing *F*_2_(*P*) will require *O*(*N* ^2^ · log⁡(*N*))  *N*-bit multiplications, obtaining an *O*(*N* ^2^ · log⁡(*N*) · Multiplication (*N*)) time complexity.

## 8. Computing the Binomial Coefficient *C*(*P*, *Q*)  (0 ≤ *Q* ≤ *P* ≤ 2^*N*^ − 1) mod⁡ 2^*N*^

Let consider the binomial coefficient *C*(*P*, *Q*) with 0 ≤ *Q* ≤ *P* ≤ 2^*N*^ − 1. In order to evaluate it modulo 2^*N*^ we will first need to compute *F*_2_(*P*), *F*_2_(*Q*), and *F*_2_(*P* − *Q*). Then, we will need to find the largest exponent exp⁡(*X*) such that 2^exp⁡(*X*)^ is a divisor of *X*!, for *X* = *P*, *Q* and *P* − *Q*. We can use (17) for this. Then, the answer will be

(30) 2exp⁡(P)−exp⁡(Q)−exp⁡(P−Q)·F2(P)  ·F2(Q)−1·F2(P−Q)−1 (mod⁡ 2N).

As discussed earlier, the multiplicative inverse of an odd number *C* modulo 2^*N*^ can be computed by using (6), using *O*(*N*)  *N*-bit multiplications. Computing exp⁡(*X*) where *X* ≤ 2^*N*^ − 1 can be performed in *O*(*N* ^2^) time (*O*(*N*) bit shifts to the right and *O*(*N*)  *N*-bit number additions). Thus, the time complexity for computing the binomial coefficient modulo 2^*N*^ is dominated by the computation of *F*_2_(*P*), *F*_2_(*Q*), and *F*_2_(*P* − *Q*).

## 9. Extensions for Computing *C*(*P*, *Q*) Modulo 2^*N*^ for *P* ≥ 2^*N*^

In order to compute a binomial coefficient *C*(*P*, *Q*) modulo 2^*N*^ for *P* ≥ 2^*N*^ (and 0 ≤ *Q* ≤ *P*), we need to make use of some variations of Lucas' theorem [5]. If *P* = *P*_1_ · 2^*N*^ + *P*_0_ and *Q* = *Q*_1_ · 2^*N*^ + *Q*_0_, then, according to [5], we have

(31) C(P,Q)  =C(P1·2N/2,Q1·2N/2)·C(P0,Q0) (mod⁡ 2N).

Thus, in order to compute *C*(*P*, *Q*) modulo 2^*N*^, we will need to evaluate *O*(log⁡(*P*)) binomial coefficients *C*(*P*′, *Q*′), where 0 ≤ *Q*′, *P*′ ≤ 2^*N*^ − 1. After the *O*(*N* ^3^ · Multiplication(*N*)) preprocessing stage, evaluating *C*(*P*, *Q*) modulo 2^*N*^ will take only *O*(*N* ^2^ · log⁡(*N*) · log⁡(*P*) · Multiplication(*N*) + log⁡^2^⁡(*P*)) time (the log^2^(*P*) factor appears when *P* ≥ 2^*N*^ because we need to perform *O*(log⁡(*P*)) divisions, each of which takes *O*(log⁡(*P*)) time because they can be implemented by shifting bits to the right, in order to obtain the *O*(log⁡(*P*)) binomial coefficients or factorials which are needed in order to compute *C*(*P*, *Q*) modulo 2^*N*^).

Other methods for reducing the computation of *C*(*P*, *Q*) modulo 2^*N*^ (for *P* ≥ 2^*N*^) to the computation of multiple (*O*(log⁡(*P*))) factorials *P*′! (of a restricted type) with *P*′ ≤ 2^*N*^ − 1 were presented in [6].

## 10. Related Work

The problem of computing binomial coefficients modulo various numbers has been widely studied in the scientific literature. In [7] properties of binomial coefficients modulo prime numbers are discussed, including Lucas' theorem, which provides a simple way of computing *C*(*P*, *Q*) modulo *R*, where *R* is a prime number. The computation of *C*(*P*, *Q*) is reduced to the computation of multiple (*O*(log⁡(*P*))) binomial coefficients *C*(*P*′, *Q*′) modulo *R*, with 0 ≤ *Q*′ ≤ *P*′ ≤ *R* − 1. In [8] the authors studied periodicity properties of the binomial coefficients modulo both prime and composite numbers. Congruence properties of binomial coefficients modulo prime powers were presented in [9], and congruence properties of products of binomial coefficients modulo composite numbers were studied in [10]. The general method of computing binomial coefficients modulo a composite number *M* is to evaluate them modulo the (maximal) prime powers which are divisors of *M* and then use the Chinese Remained Theorem [11] in order to obtain the result modulo *M* (as observed in [10]), but in [10] a more direct study of these values is performed (i.e., the Chinese Remainder Theorem is not used). Properties of the residues of binomial coefficients and their products modulo prime powers were studied in [12].

An algorithm for computing binomial coefficients modulo prime powers (for any prime) was presented in [6]. The algorithm takes *O*(log⁡^2^⁡(*P*) + *v* ^4^ · log⁡(*P*) · log⁡(*u*) + *v* ^4^ · *u* · log⁡^3^⁡(*u*)) time for computing *C*(*P*, *Q*) modulo *u* ^*v*^, where *u* is a prime number (this time complexity was stated in [6], but a sufficiently detailed complexity analysis was not provided). When *u* = 2 and *v* = *N*, this reduces to *O*(log⁡^2^⁡(*P*) + *N* ^4^ · log⁡(*P*) + *N* ^4^). If we consider Multiplication(*N*) = *O*(*N* ^2^), our algorithm takes *O*(*N* ^5^) time for preprocessing and *O*(*N* ^4^ · log⁡(*N*) · log⁡(*P*) + log⁡^2^⁡(*P*)) in order to compute *C*(*P*, *Q*) in the general case. These time complexities are slightly worse than the ones obtained in [6]. However, when considering Multiplication(*N*) = *O*(*N* · log⁡(*N*) · log⁡(log⁡(*N*))) [1, 2], we obtain an *O*(*N* ^4^ · log⁡(*N*) · log⁡(log⁡(*N*))) time complexity for the preprocessing stage and an *O*(*N* ^3^ · log⁡^2^⁡(*N*) · log⁡⁡(log⁡(*N*)) · log⁡(*P*) + log⁡^2^⁡(*P*)) time complexity for actually computing the binomial coefficient modulo 2^*N*^. In this case our time complexities are slightly better than the ones presented in [6] (when log⁡(*P*) > log⁡(*N*) · log⁡⁡(log⁡(*N*))). However, it is not clear which time complexity for the multiplication of two *N*-bit numbers was considered in [6].

Reference [13] uses sums of binomial coefficients modulo 2 when obtaining results related to the Garsia entropy. Binomial coefficients also occur in other equations regarding information theory formulas (e.g., [14, 15]). Binomial coefficients also have applications in many other areas (e.g., statistics [16], binomial distribution [17], and Chebyshev polynomials [18]).

## 11. Conclusions and Future Work

In this paper I presented a novel efficient algorithm for computing binomial coefficients modulo 2^*N*^. The algorithm consists of a preprocessing stage, after which any number of binomial coefficients can be computed modulo 2^*N*^ (or modulo any number 2^*Q*^ with 0 ≤ *Q* ≤ *N*).

The time complexity of the presented algorithm is comparable with that of state-of-the-art algorithms for computing binomial coefficients modulo prime powers [6]. In fact, the time complexity of the algorithm presented in this paper is slightly better than that of the algorithm presented in [6], but since a sufficiently detailed analysis of the time complexity in [6] is not provided by the authors, it is not clear if the algorithm from [6] cannot be improved any further. In any case, because the algorithm presented in this paper consists of a preprocessing stage, a slightly worse preprocessing time complexity (in some cases) could be balanced by computing multiple binomial coefficients (when the preprocessing stage, which is the bottleneck, is run only once).

When computing a small number of binomial coefficients (e.g., just one), the bottleneck of the algorithm (in the preprocessing stage) consists of the computation of sums of products of elements of subsets having sizes 0 to *N* (described in Section 5). That step requires *N* ^3^ multiplications of *N*-bit numbers, while all the other steps need fewer multiplications (and one of the steps requires *N* ^3^ additions of *N*-bit numbers). If the values SSP(*P*, *Q*) defined in Section 5 could be computed faster, the algorithm presented in this paper could be considerably improved. In future work, I intend to study the problem of computing the SSP(*P*, *Q*) values in a more efficient manner.
